# Supplementary material for: Entangling Credit and Funding Shocks in Interbank Markets
Source: PLoS One. 2016 Aug 25;11(8):e0161642. doi: 10.1371/journal.pone.0161642 (PMC4999134; doi:10.1371/journal.pone.0161642)
Supplement: S1 Fig — For each bank i, the number of outgoing links is the number of debtors of i, namely its out-degree, and is defined as kiout=∑jaij. Analogously, the number of incoming links is the number of creditors of i, namely its in-degree, defined as kiin=∑jaji. Using Eq (2) we get 〈kiout〉=∑jpij=∑j(AiLj)/(z-1+AiLj) and 〈kiin〉=∑jpji=∑j(AjLi)/(z-1+AjLi). (PDF) [file pone.0161642.s001.pdf]

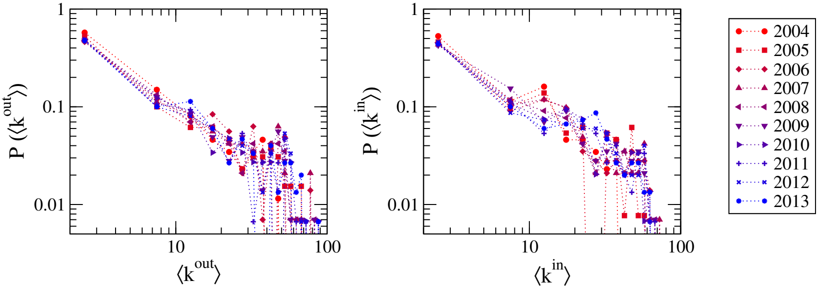

S1 Fig. Probability distributions for the average number of outgoing and incoming connections of banks, for years from 2004 to 2013. For each bank i, the number of outgoing links is the number of debtors of i, namely its *out-degree*, and is defined as $k_i^{out} = \sum_j a_{ij}$. Analogously, the number of incoming links is the number of creditors of i, namely its *in-degree*, defined as $k_i^{in} = \sum_j a_{ji}$. Using eq. (2) of the main text, we get $\langle k_i^{out} \rangle = \sum_j p_{ij} = \sum_j (A_i\, L_j)/(z^{-1} + A_i\, L_j)$ and $\langle k_i^{in} \rangle = \sum_j p_{ji} = \sum_j (A_j\, L_i)/(z^{-1} + A_j\, L_i)$.
