# Supplementary figures and images for: Entangling Credit and Funding Shocks in Interbank Markets
Source: PLoS One. 2016 Aug 25;11(8):e0161642. doi: 10.1371/journal.pone.0161642 (PMC4999134; doi:10.1371/journal.pone.0161642)

S2 File. Slideshow with results of group DS Rank  
for years from 2004 to 2013.

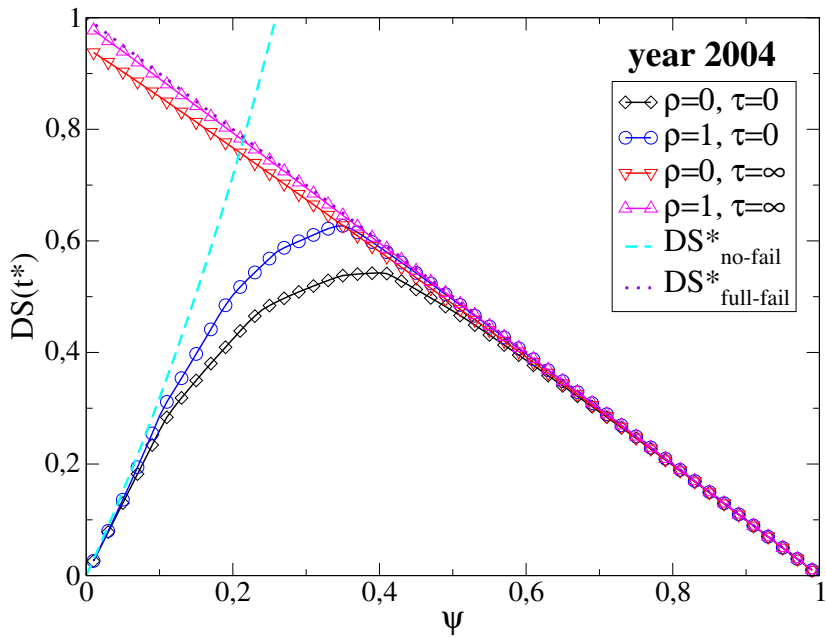

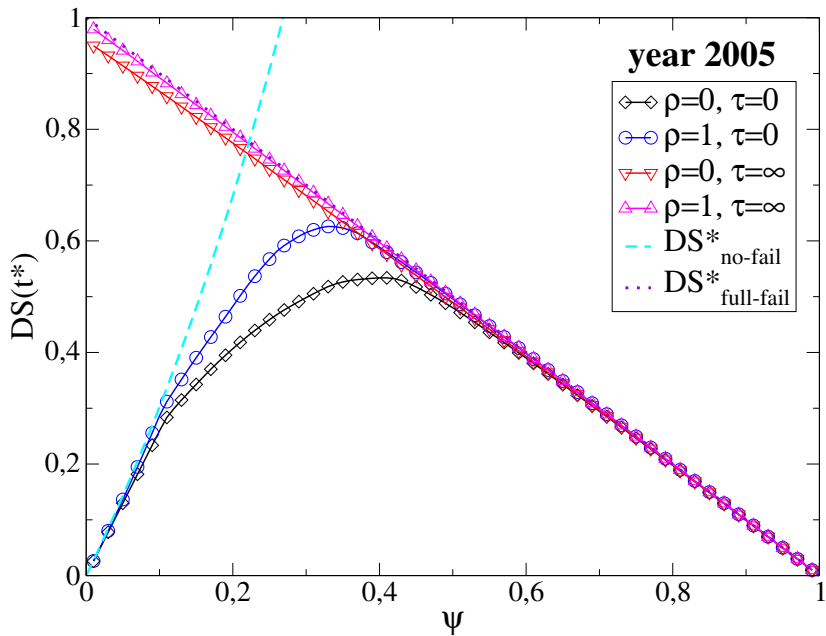

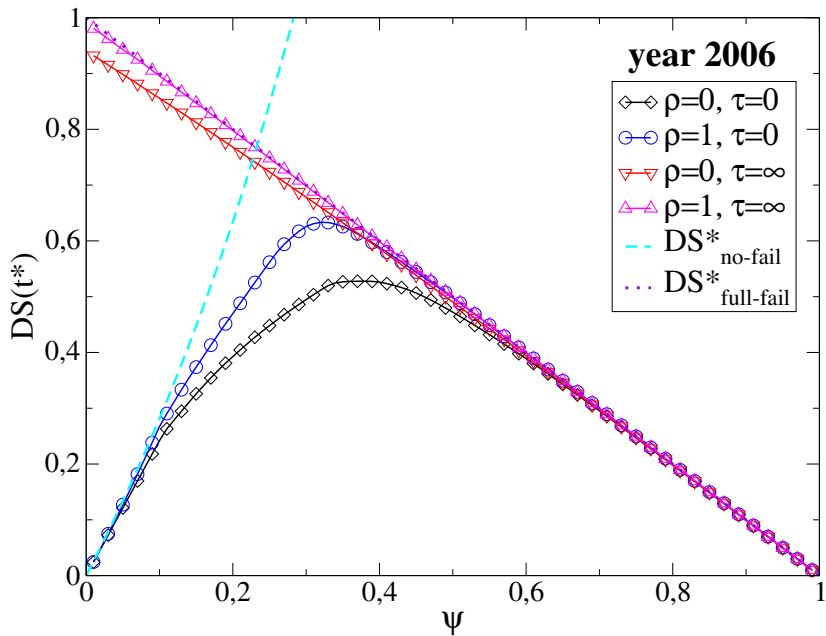

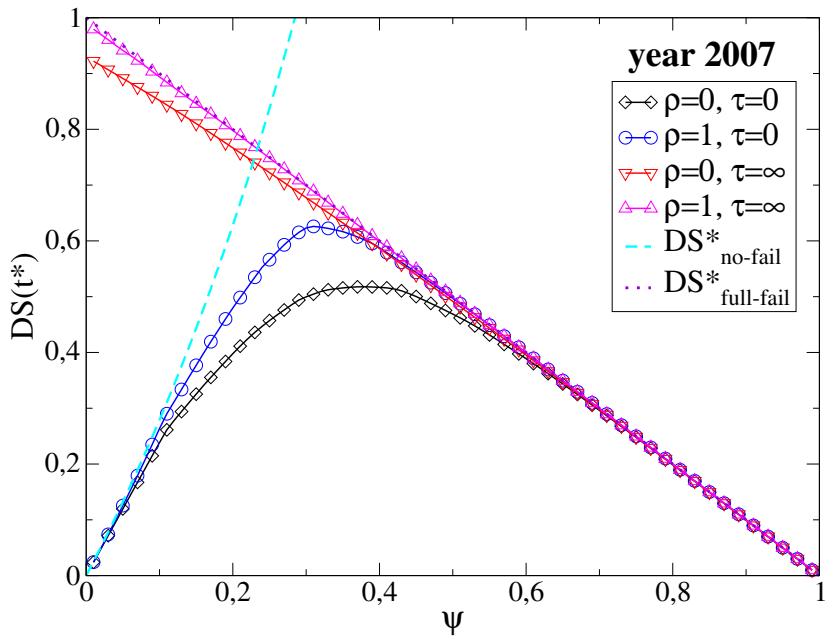

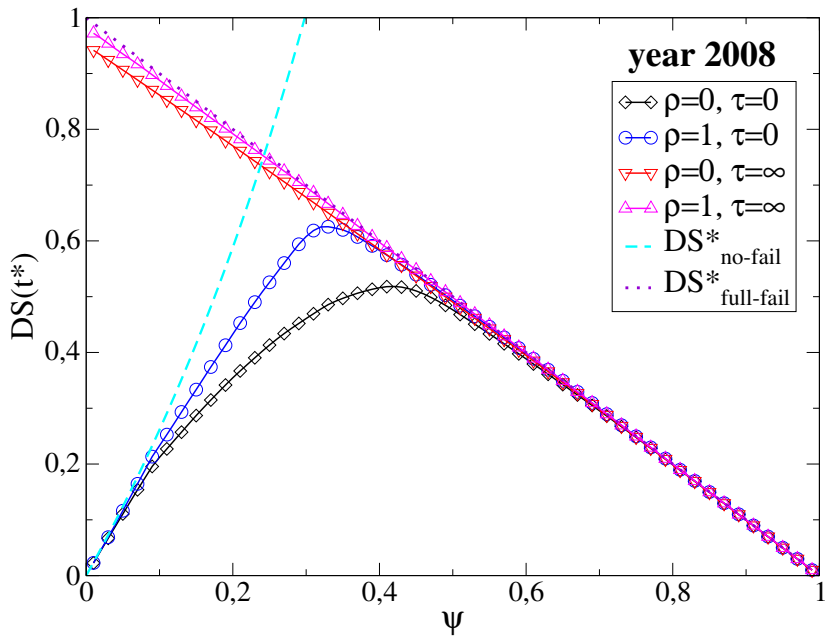

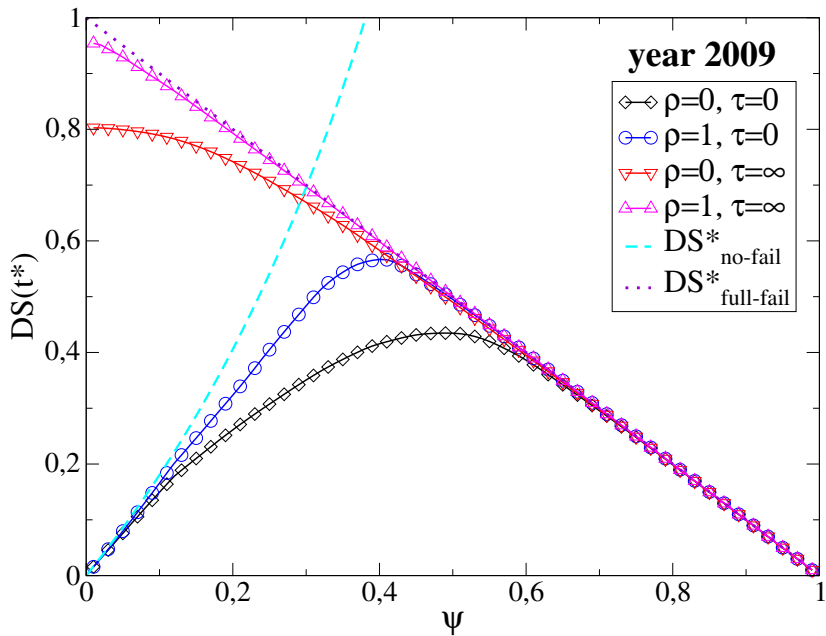

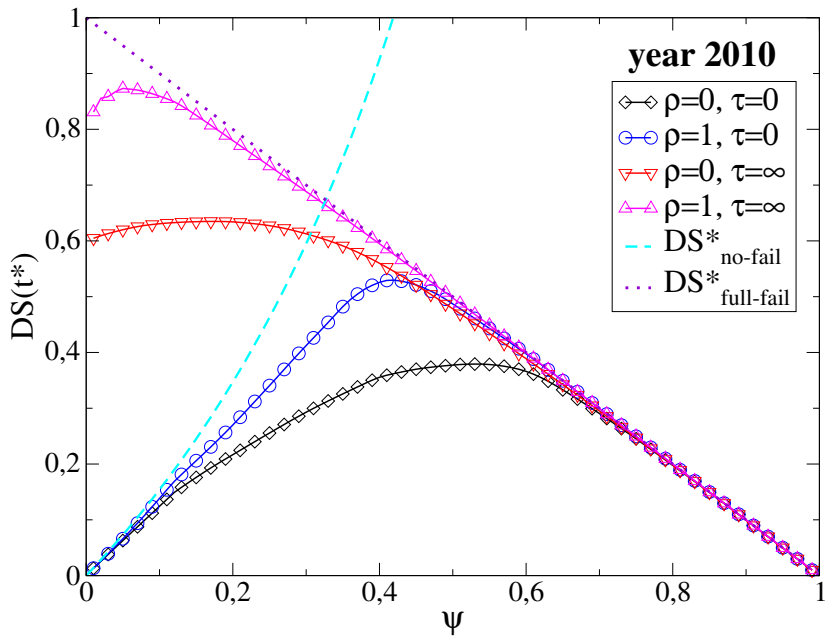

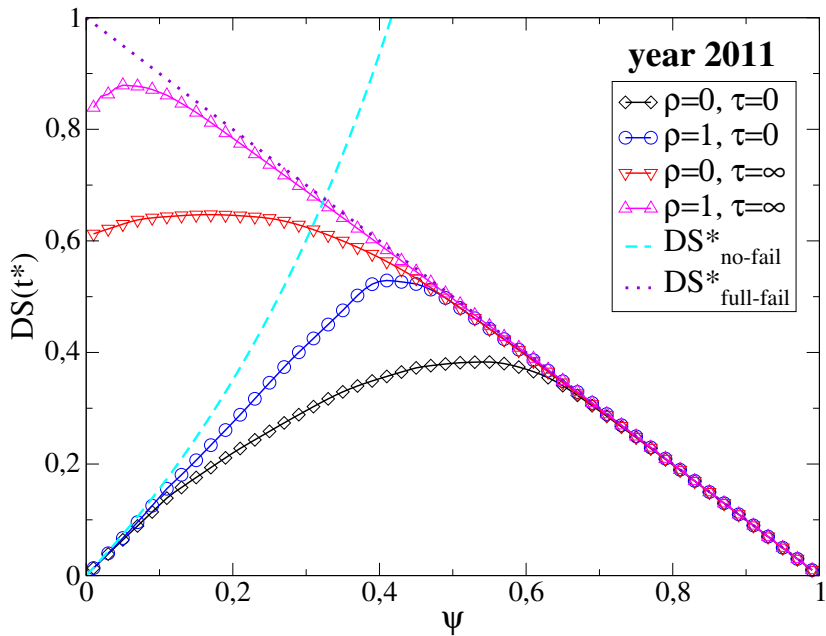

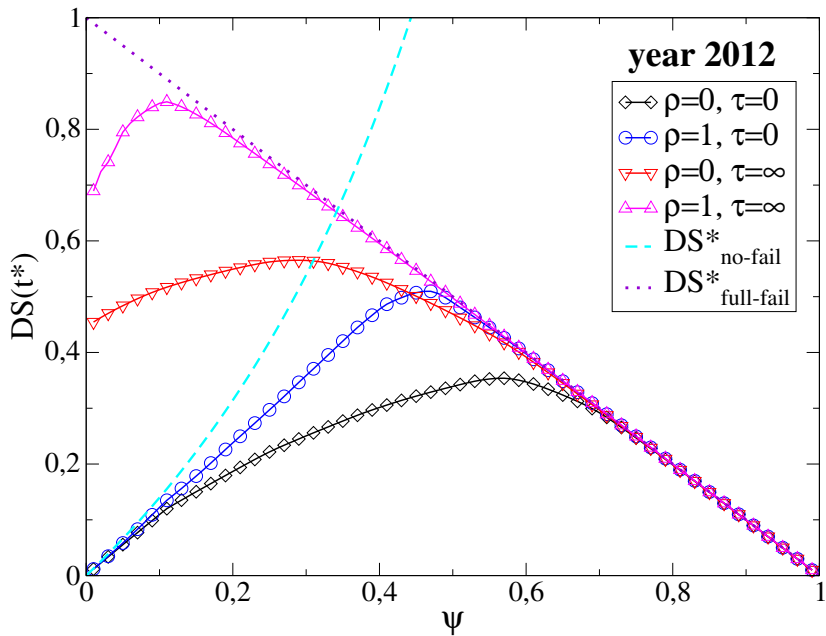

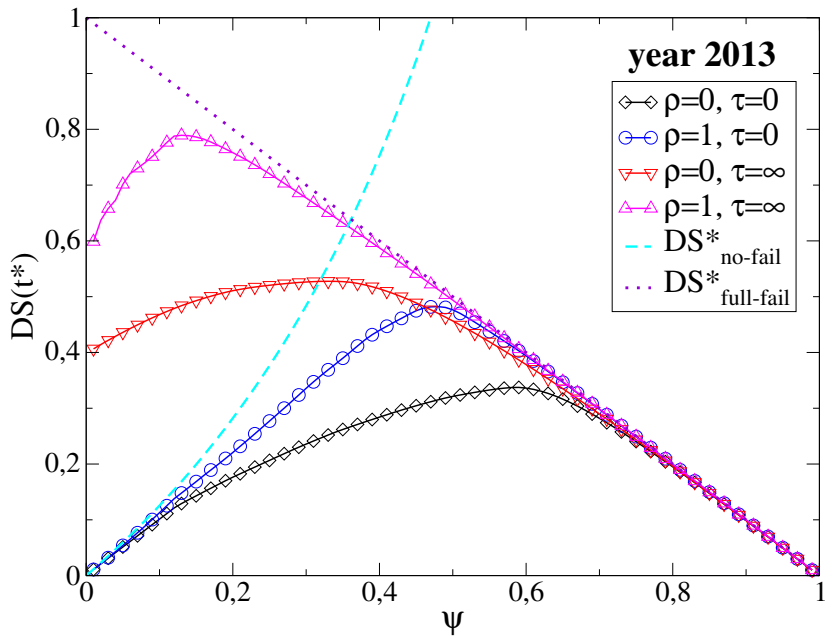

Supplement: S1 File — (PDF) [file pone.0161642.s002.pdf]
