# Supplementary material for: Entangling Credit and Funding Shocks in Interbank Markets
Source: PLoS One. 2016 Aug 25;11(8):e0161642. doi: 10.1371/journal.pone.0161642 (PMC4999134; doi:10.1371/journal.pone.0161642)
Supplement: S2 File — (PDF) [file pone.0161642.s003.pdf]

S2 File. Slideshow with scatter plots of impact and vulnerability of individual banks for years from 2004 to 2013.

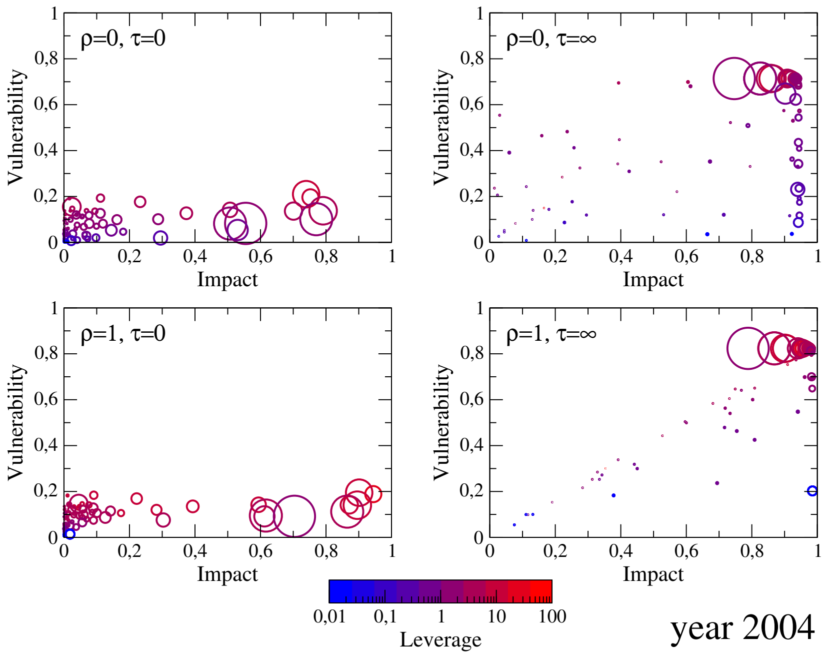

ρ=0, τ=0
ρ=0, τ=∞
ρ=1, τ=0
ρ=1, τ=∞
Vulnerability
Impact
0
0,2
0,4
0,6
0,8
1
Leverage
0,01
0,1
1
10
100
year 2004

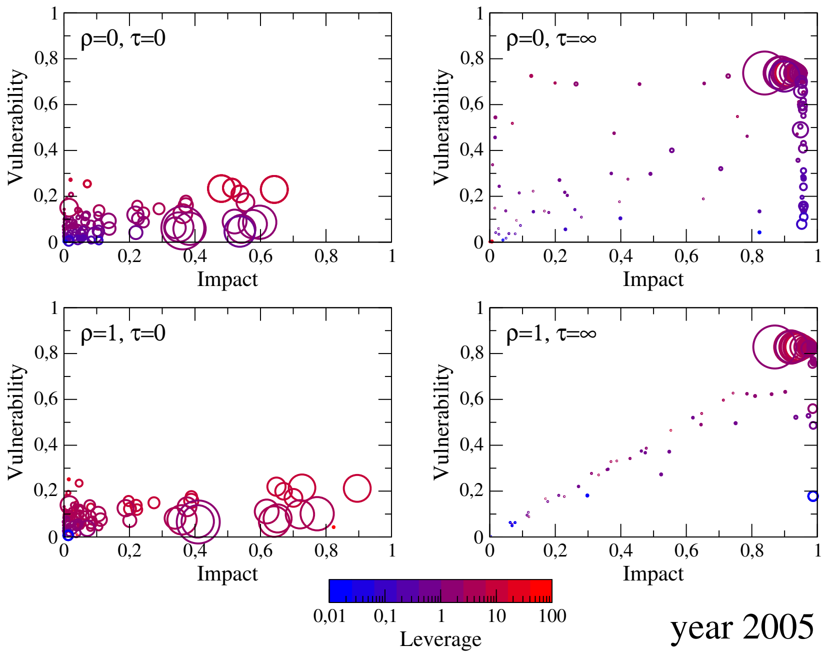

ρ=0, τ=0
ρ=0, τ=∞
ρ=1, τ=0
ρ=1, τ=∞
Vulnerability
Impact
0
0,2
0,4
0,6
0,8
1
0,01
0,1
1
10
100
Leverage
year 2005

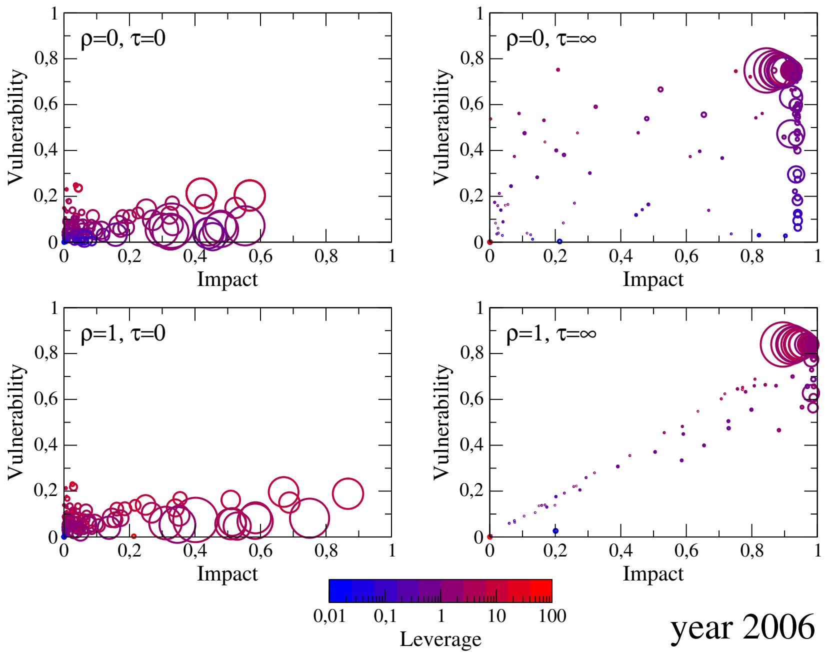
ρ=0, τ=0
ρ=0, τ=∞
ρ=1, τ=0
ρ=1, τ=∞
Vulnerability
Impact
0
0,2
0,4
0,6
0,8
1
0,01
0,1
1
10
100
Leverage
year 2006

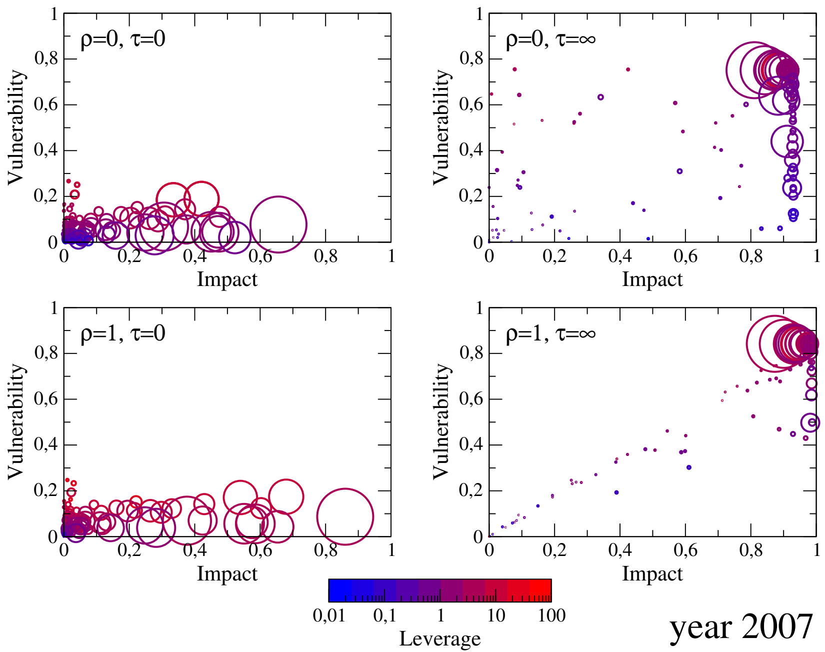
ρ=0, τ=0
ρ=0, τ=∞
ρ=1, τ=0
ρ=1, τ=∞
Vulnerability
Impact
0
0,2
0,4
0,6
0,8
1
Leverage
0,01
0,1
1
10
100
year 2007

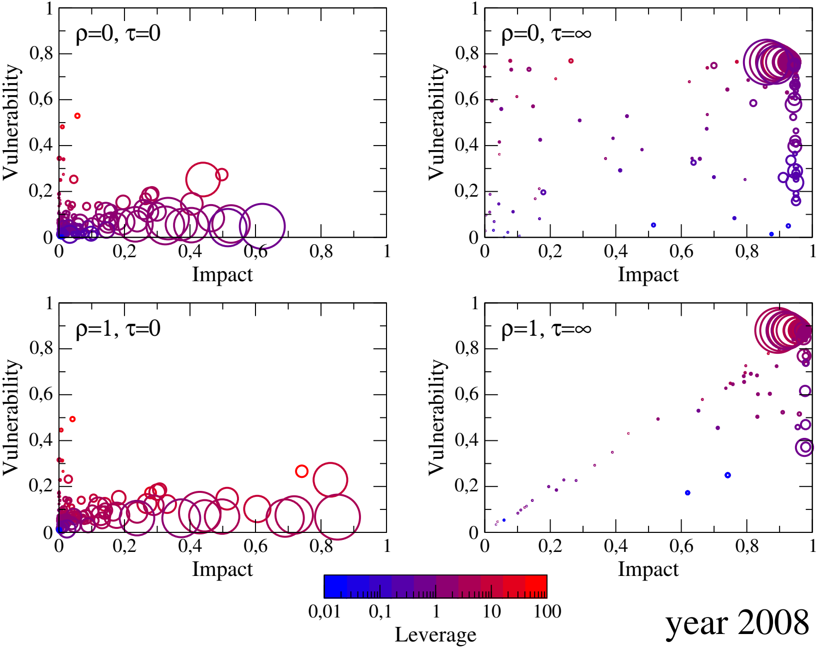

ρ=0, τ=0
ρ=0, τ=∞
ρ=1, τ=0
ρ=1, τ=∞
Vulnerability
Impact
0
0,2
0,4
0,6
0,8
1
0,01
0,1
1
10
100
Leverage
year 2008

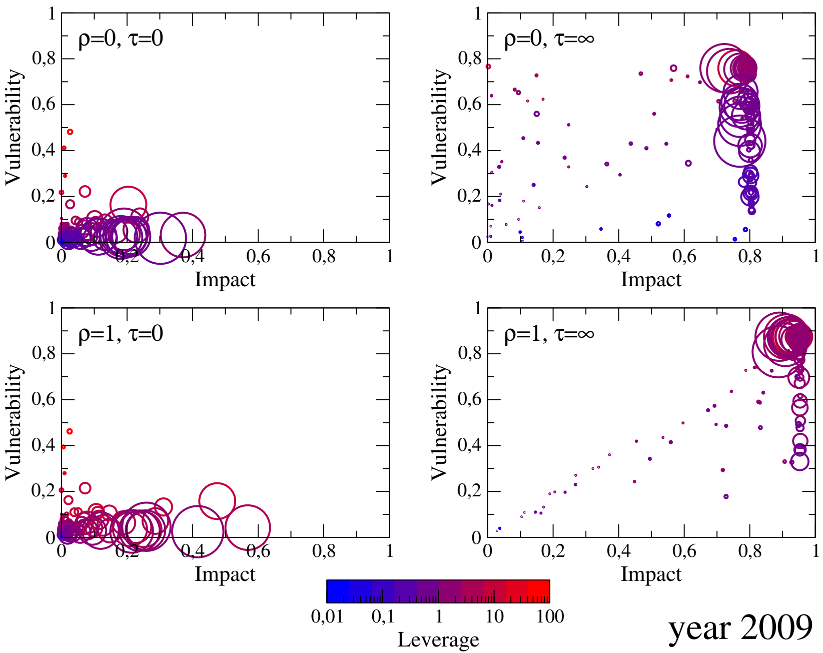

ρ=0, τ=0
ρ=0, τ=∞
ρ=1, τ=0
ρ=1, τ=∞
Vulnerability
Impact
0
0,2
0,4
0,6
0,8
1
Leverage
0,01
0,1
1
10
100
year 2009

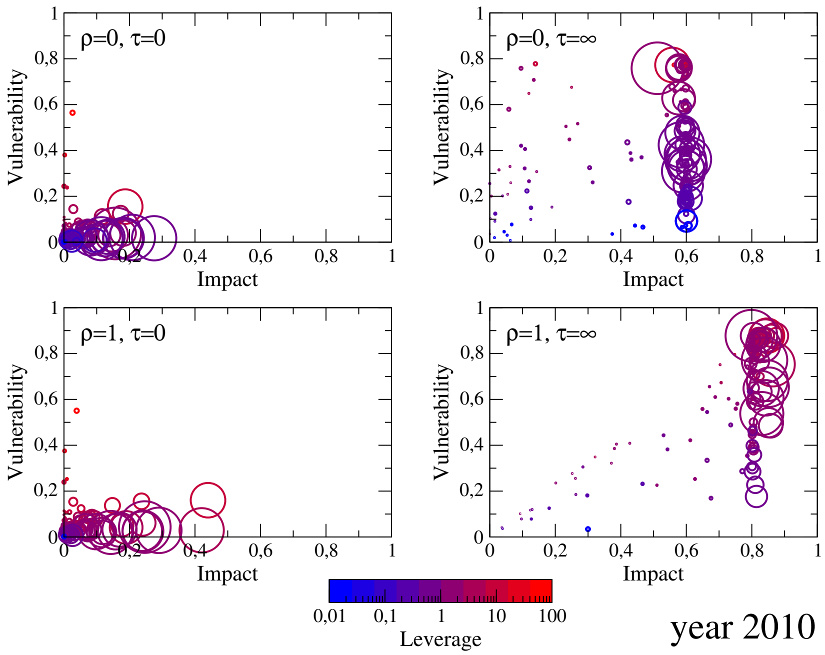
ρ=0, τ=0
ρ=0, τ=∞
ρ=1, τ=0
ρ=1, τ=∞
Vulnerability
Impact
0
0,2
0,4
0,6
0,8
1
0,01
0,1
1
10
100
Leverage
year 2010

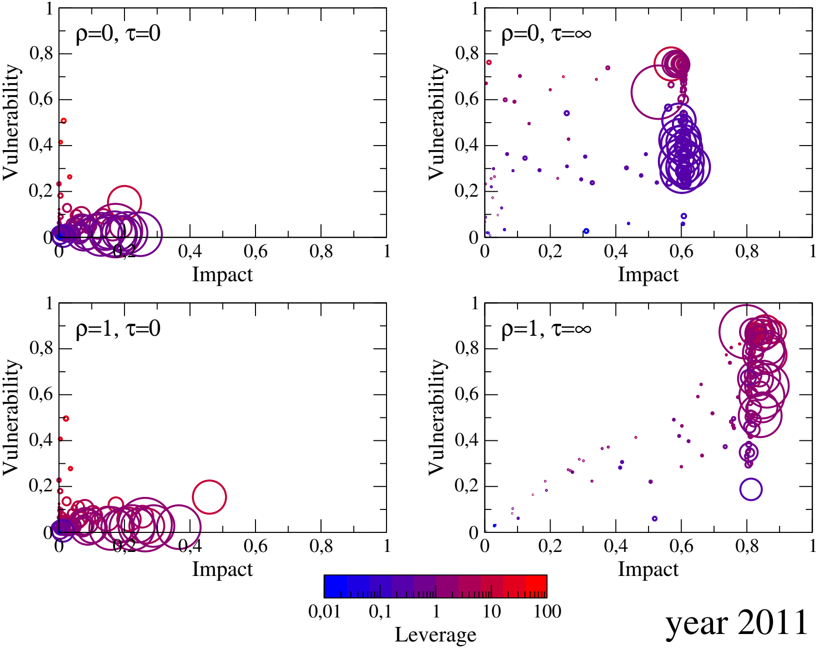

ρ=0, τ=0
ρ=0, τ=∞
ρ=1, τ=0
ρ=1, τ=∞
Vulnerability
Impact
0
0,2
0,4
0,6
0,8
1
0,01
0,1
1
10
100
Leverage
year 2011

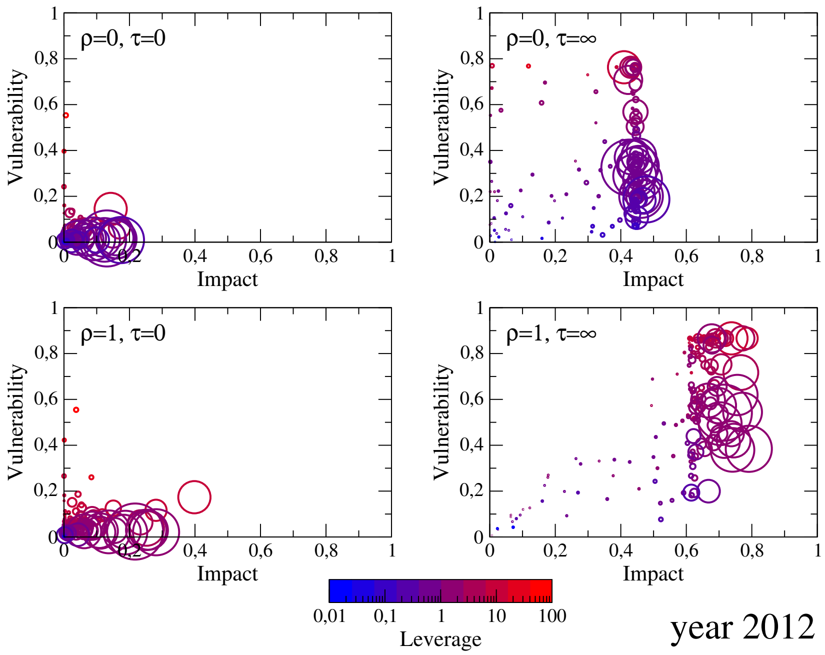
ρ=0, τ=0
ρ=0, τ=∞
ρ=1, τ=0
ρ=1, τ=∞
Vulnerability
Impact
0
0,2
0,4
0,6
0,8
1
Leverage
0,01
0,1
1
10
100
year 2012

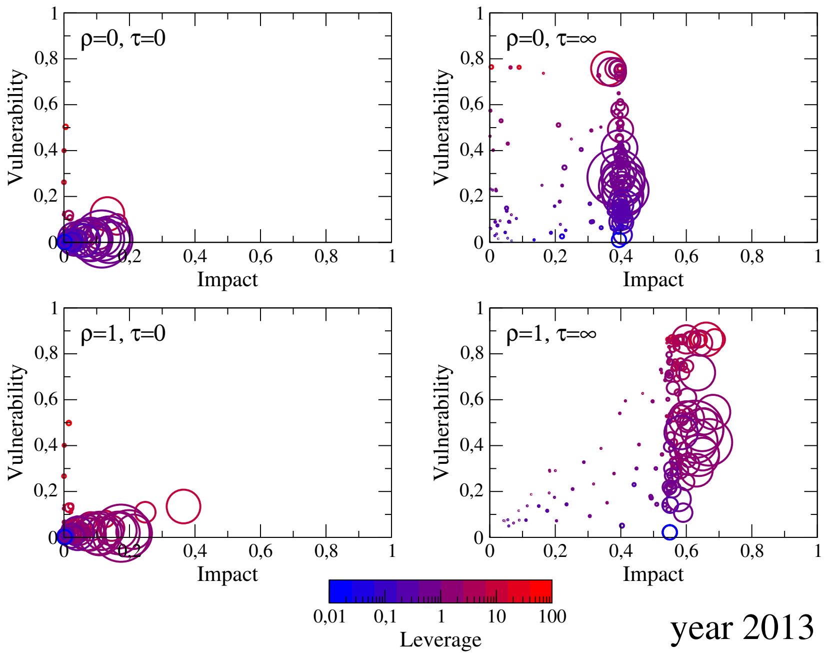
ρ=0, τ=0
ρ=0, τ=∞
ρ=1, τ=0
ρ=1, τ=∞
Vulnerability
Impact
0
0,2
0,4
0,6
0,8
1
0,01
0,1
1
10
100
Leverage
year 2013
